# Immunogenicity of Botulinum Toxin Type A in Different Clinical and Cosmetic Treatment, a Literature Review

**DOI:** 10.3390/life14101217

**Published:** 2024-09-24

**Authors:** Kar Wai Alvin Lee, Lisa Kwin Wah Chan, Angela Wai Kay Lee, Cheuk Hung Lee, Jovian Wan, Kyu-Ho Yi

**Affiliations:** 1 EverKeen Medical Centre, Hong Kong; 2 The Skin Oracle, Hong Kong; 3 Asia Pacific Aesthetic Academy, Hong Kong; 4 Division in Anatomy and Developmental Biology, Department of Oral Biology, Human Identification Research Institute, BK21 FOUR Project, Yonsei University College of Dentistry, 50-1, Seoul 03722, Republic of Korea; 5 Maylin Clinic (Apgujeong), Seoul 03722, Republic of Korea

**Keywords:** botulinum toxins, type A, immunogenicity, vaccine, neutralizing antibodies, treatment failure, cosmetic techniques

## Abstract

Background: Botulinum toxin type A is widely utilized for both therapeutic and aesthetic purposes, yet concerns regarding its immunogenicity have raised issues related to treatment failure and adverse reactions. Objective: This review aims to evaluate the immunogenicity of commercially available botulinum toxin type A products across various clinical indications and identify the risk factors associated with antibody formation. Methods: A comprehensive search of electronic databases was conducted to find studies that investigated the immunogenicity of botulinum toxin type A in patients treated for different conditions. The studies were classified based on the Oxford Center for Evidence-Based Medicine’s evidence hierarchy. Results: The overall incidence of neutralizing antibody formation with botulinum toxin type A treatment is relatively low. However, it varies depending on the indication and is influenced by factors such as the frequency of injections and the cumulative dose. The total cumulative dose and the number of treatment cycles are critical factors in determining the risk of developing antibodies against botulinum toxin type A. Conclusion: This literature review highlights that the immunogenicity of botulinum toxin type A products differs across indications, with repeated injections posing a significant risk for the formation of neutralizing antibodies. The findings underscore the need for further research to better understand antibody formation mechanisms and to develop strategies that minimize their impact on treatment efficacy.

## 1. Introduction

Botulinum toxin treatment is the most popular non-surgical cosmetic procedure, widely used for various therapeutic and aesthetic applications. Despite its effectiveness, the effects of botulinum toxin are transient, necessitating repeated injections to maintain the desired outcomes.

However, these repeated injections can trigger an immune response in the body. As botulinum toxin functions as an antigen, the immune system may gradually recognize and eliminate it, potentially leading to therapeutic failure [1,2]. This immune response is particularly problematic because it can interfere with the toxin’s efficacy over time, making it less effective or even completely ineffective in some cases. The specific actions of the complexing proteins and core neurotoxin protein that make up active botulinum toxin are still not fully understood, further complicating the issue [3].

The development of neutralizing antibodies (nAbs) is a significant concern associated with repeated botulinum toxin treatments. These specialized antibodies are produced by the immune system in response to the toxin and are capable of directly neutralizing its biological activity (Simpson, 2013). Neutralizing antibodies bind to specific regions on the botulinum toxin molecule, preventing it from interacting with its target receptors on nerve cells. This blockade inhibits the toxin’s intended action of reducing muscle activity by stopping the release of acetylcholine at the neuromuscular junction. The production of these antibodies is closely linked to the complexing proteins that accompany the core neurotoxin protein in botulinum toxin preparations [4]. The presence of nAbs can lead to secondary treatment failure in which the therapeutic effects diminish, requiring higher doses or rendering the treatment ineffective altogether [2,4].

To manage the risk of therapeutic failure due to neutralizing antibodies, various methods are employed to detect these antibodies in patients undergoing botulinum toxin therapy. The mouse protection assay (MPA) and the mouse neutralization assay (MNA) are considered the gold standards for detecting nAbs due to their ability to directly measure the functional neutralization of the toxin [3]. Enzyme-linked immunosorbent assays (ELISA) offer a faster and more accessible alternative but may detect both neutralizing and non-neutralizing antibodies, potentially leading to false positives [4]. The ninhydrin sweat test is another method explored for its simplicity and non-invasiveness. The early detection of neutralizing antibodies is crucial for adjusting treatment plans to prevent or mitigate the effects of immunoresistance, ensuring the continued efficacy of botulinum toxin treatments [5]. 

**Figure 1:**
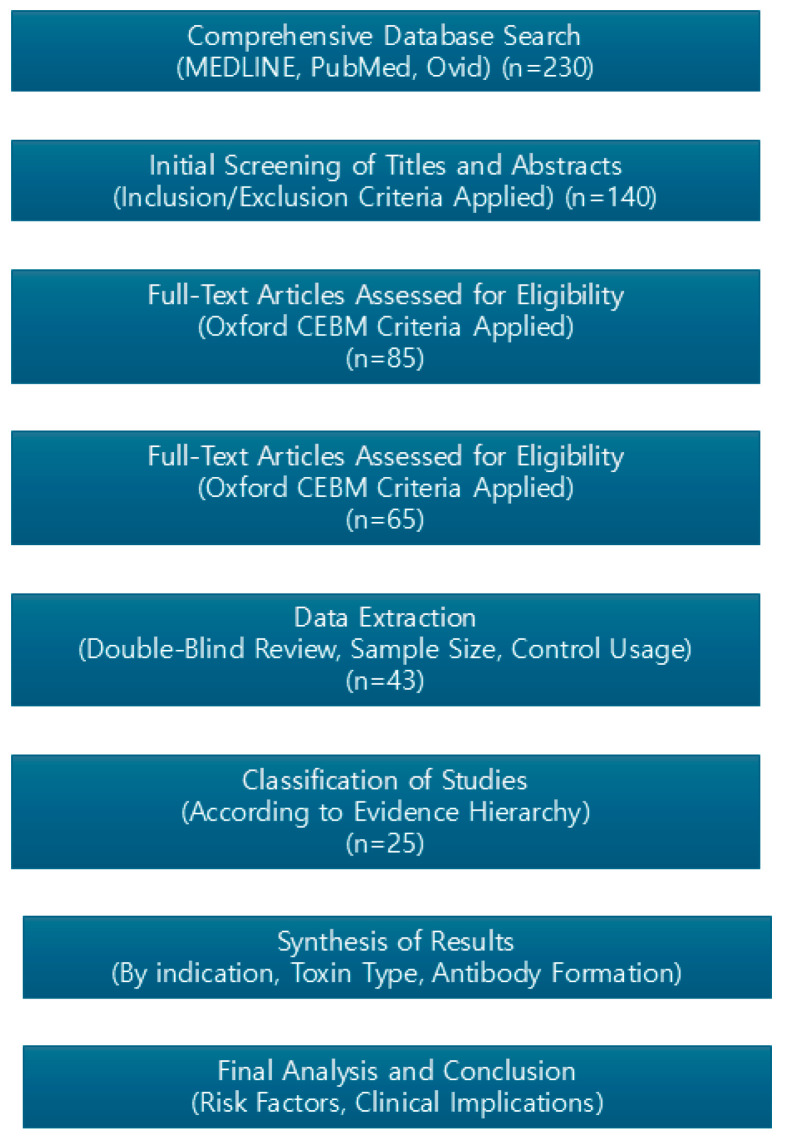
The flow chart of the study design.

**Figure 2:**
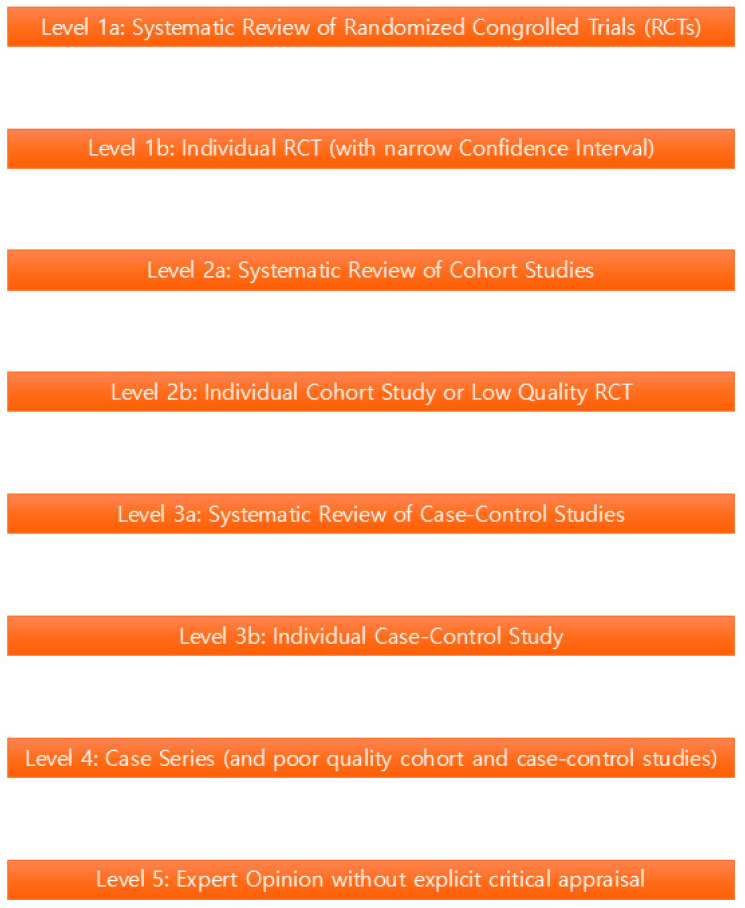
The diagram representing the Oxford Center for Evidence-Based Medicine (CEBM)’s evidence hierarchy. It visually outlines the classification of studies from the highest level of evidence (systematic reviews of RCTs) down to the lowest level (expert opinion without explicit critical appraisal).

Keywords including “Immunogenicity”, “Neutralizing Antibodies”, “Onabotulinum A”, “Abobotulinum A”, “Incobotulinum A”, and “Botulinum Toxin Type A” were searched in the MEDLINE, PubMed, and Ovid databases for relevant studies published on clinical trials, diagnosis, and treatment (Figure 1). Some papers were further reviewed based on double-blind approach usage, sample size, control usage, randomization usage, and objective endpoint measurements. All studies were classified according to the Oxford Center for Evidence-Based Medicine’s evidence hierarchy (Figure 2).

## 2. Systematic Reviews and Meta-Analyses

Rahman et al. [6] conducted a systematic search of electronic databases to identify studies that assessed the immunogenicity of botulinum toxin type A in patients receiving treatment for different indications. The inclusion criteria were human studies, published in English, investigating the immunogenicity of botulinum toxin type A, including patients with different indications. The search yielded 24 studies that met the inclusion criteria. The studies were evaluated for their quality using the Newcastle-Ottawa scale (NOS).

The results showed that the overall pooled incidence of neutralizing antibody formation was 10.1% (95% CI: 5.1–17.3%). The incidence of neutralizing antibody formation varied across indications, ranging from 2.1% in cervical dystonia to 26.7% in blepharospasm. The incidence of neutralizing antibody formation was higher in patients receiving repeated injections (14.5% vs. 4.5%), with the duration of treatment not significantly affecting neutralizing antibody formation. The authors concluded that botulinum toxin type A immunogenicity varies across different indications, and repeated injections may increase the risk of neutralizing antibody formation. However, the overall incidence of neutralizing antibody formation remains relatively low, and no severe adverse events were reported (Level IIa).

Jankovic et al. [7] presented a meta-analysis of neutralizing antibody formation with onabotulinumtoxinA (Botox) treatment across multiple indications. Botox is a botulinum neurotoxin type A used for various therapeutic and aesthetic applications, including blepharospasm, strabismus, cervical dystonia, and spasmodic torticollis.

The authors conducted a meta-analysis of data from global registration studies to assess the incidence of neutralizing antibody formation with Botox treatment. The inclusion criteria were studies investigating neutralizing antibody formation with Botox, including patients with different indications, and published in English.

The search yielded 34 studies that met the inclusion criteria. The studies were evaluated for their quality using the Cochrane risk of bias tool. The results showed that the overall pooled incidence of neutralizing antibody formation was 10.3% (95% CI: 7.5–13.2%). The incidence of neutralizing antibody formation varied across indications, ranging from 4.4% in blepharospasm to 23.1% in spasmodic torticollis. The incidence of neutralizing antibody formation was higher in patients receiving repeated injections (14.4% vs. 6.5%), the duration of treatment did not significantly affect neutralizing antibody formation, and the risk of neutralizing antibody formation was higher in patients receiving higher doses of Botox (OR = 1.05, 95% CI: 1.02–1.08).

The authors concluded that the overall incidence of neutralizing antibody formation with Botox treatment is relatively low, but it varies across different indications and is influenced by factors such as repeated injections and dose (Level Ia).

## 3. Clinical Studies on Immunogenicity and Treatment Response 

Kranz et al. [8] investigated the presence of neutralizing antibodies against botulinum toxin type A in patients with dystonia who continued to respond well to treatment. Neutralizing antibodies are immune system proteins that can bind to and neutralize the effect of botulinum toxin type A, reducing its efficacy.

The study included 50 patients with dystonia who had been treated with botulinum toxin type A for at least 6 months. Blood samples were collected from each patient and tested for the presence of neutralizing antibodies using a functional assay. The patients were also evaluated clinically for their response to botulinum toxin type A treatment.

The results showed that 44% of patients (22/50) had detectable levels of neutralizing antibodies against botulinum toxin type A. However, surprisingly, all 22 patients with neutralizing antibodies continued to respond well to botulinum toxin type A treatment, with no significant decrease in efficacy. The researchers also found that the presence of neutralizing antibodies was not associated with a lower dose requirement for botulinum toxin type A or a shorter duration of effect.

The study’s findings suggest that the presence of neutralizing antibodies against botulinum toxin type A does not necessarily predict a loss of response to treatment in patients with dystonia. The authors propose that the immune system’s ability to produce neutralizing antibodies may be overwhelmed by the large amount of toxin used in treatment, or that the toxin’s binding sites on the muscle fibers may be altered, making it more difficult for the antibodies to bind and neutralize the toxin (Level IIc).

Wu et al. [9] evaluated the safety and efficacy of onabotulinumtoxinA for the treatment of crow’s feet lines in Chinese subjects in a randomized, double-blind, placebo-controlled study. The study aimed to investigate the immunogenicity of botulinum toxin type A in this population, and 120 Chinese patients with moderate to severe crow’s feet lines were enrolled. Participants were randomly assigned to receive either onabotulinumtoxinA or a placebo. The primary outcome measure was the severity of crow’s feet lines at 30 days post-treatment, as assessed by a blinded investigator.

The results showed that onabotulinumtoxinA significantly reduced the severity of crow’s feet lines compared to the placebo group, with a mean change score of −1.43 ± 0.27 (*p* < 0.001). Moreover, the treatment was well-tolerated, with no serious adverse events reported. Antibody formation against botulinum toxin type A was evaluated at baseline, 60 days, and 180 days post-treatment. Antibody titers were significantly increased in the placebo group (*p* < 0.01), indicating no neutralizing antibodies detected in the placebo group. However, the antibody titers in the onabotulinumtoxinA-treated group remained stable throughout the study period, suggesting that the treatment did not induce significant immunogenicity (Level IIb).

Wu et al [9]. evaluated the efficacy and safety of botulinum toxin type A for the treatment of glabellar lines in Chinese patients. The study was a double-blind, randomized, placebo-controlled trial that aimed to investigate the immunogenicity of BTX-A in this population. A total of 120 patients with moderate to severe glabellar lines were randomly assigned to receive either botulinum toxin type A or a placebo. The primary outcome measure was the severity of glabellar lines at 30 days post-treatment, as assessed by a blinded investigator.

The results showed that botulinum toxin type A significantly improved the severity of glabellar lines compared to the placebo group, with a mean change score of −1.43 ± 0.27 (*p* < 0.001). The treatment was well-tolerated, with no serious adverse events reported. Antibody formation against botulinum toxin type A was evaluated at baseline, 60 days, and 180 days post-treatment. Antibody titers were significantly increased in the placebo group (*p* < 0.01), indicating a natural response to botulinum toxin type A exposure. However, the antibody titers in the botulinum toxin type A treated group remained stable throughout the study period, suggesting that the treatment did not induce significant immunogenicity. The study also evaluated the persistence of the effect over time, showing that the improvement in glabellar lines was maintained for up to 180 days after treatment (Level IIa).

Harii et al. [10] compared the efficacy and safety of two different doses of botulinum toxin type A for the treatment of glabellar lines in Japanese subjects in their study. It was a double-blind, randomized, placebo-controlled trial that aimed to evaluate the immunogenicity of botulinum toxin type A in this population.

A total of 120 Japanese patients with moderate to severe glabellar lines were randomly assigned to receive either a low dose (25 units), a high dose (50 units), or a placebo. The primary outcome measure was the severity of glabellar lines at 30 days post-treatment, as assessed by a blinded investigator.

The results showed that both the low-dose and high-dose botulinum toxin type A groups significantly improved the severity of glabellar lines compared to the placebo group, with mean change scores of −1.27 ± 0.29 and −1.62 ± 0.32, respectively (*p* < 0.001). The treatment was well-tolerated, with no serious adverse events reported. Antibody formation against botulinum toxin type A was evaluated at baseline, 60 days, and 180 days post-treatment. Antibody titers were significantly increased in the placebo group (*p* < 0.01), indicating a natural response to botulinum toxin type A exposure. However, the antibody titers in both the low-dose and high-dose botulinum toxin type A groups remained stable throughout the study period, suggesting that neither dose induced significant immunogenicity. The study also evaluated the duration of effect over time, showing that the improvement in glabellar lines was maintained for up to 180 days after treatment (Level IIa).

Coleman et al. [11] reported the results of a study investigating the development of immunoresistance in patients with cervical dystonia who received abobotulinumtoxinA therapy. Immunoresistance is a phenomenon where the body develops antibodies that neutralize the effect of abobotulinumtoxinA, reducing its efficacy over time.

The study included 44 patients with cervical dystonia who received abobotulinumtoxinA injections every 12 weeks for at least 24 weeks. Serum samples were collected at baseline and after each injection to test for the presence of antibodies against abobotulinumtoxinA using a radioimmunoprecipitation assay.

The results showed that 25% of patients developed immunoresistance after a median of 16 weeks after the first injection. The development of immunoresistance was associated with a significant decrease in the clinical response to abobotulinumtoxinA, with a mean reduction in the Toronto Western Spasmodic Torticollis Rating Scale (TWSTRS) score of 24% compared to baseline. The authors found that patients who developed immunoresistance had higher levels of antibodies against abobotulinumtoxinA at baseline compared to those who did not develop immunoresistance. Additionally, the development of immunoresistance was associated with a longer duration of treatment and a higher total dose of abobotulinumtoxinA received.

The study concluded that immunoresistance is a significant issue in cervical dystonia patients receiving abobotulinumtoxinA therapy and that monitoring for antibodies against abobotulinumtoxinA may be necessary to identify patients at risk of developing immunoresistance. The authors suggest that alternative treatments or rotation between different abobotulinumtoxinA products may be necessary to maintain efficacy in patients who develop immunoresistance (Level IIb).

Oshima et al. [12] reported on the antibody response to botulinum toxin type A in children with spastic equinus foot due to cerebral palsy. The study aimed to compare the antibody response to botulinum toxin type A in children treated with two different injection schedules. It included 44 children with spastic equinus foot who were randomized to receive either a standard treatment schedule (every 3–4 months) or an extended treatment schedule (every 6–8 months). The children received a total of 12 injections over a period of 24 months.

The results showed that the overall antibody response to botulinum toxin type A was significantly higher in the extended treatment group compared to the standard treatment group (60.9% vs. 25.9%, respectively). The median time to develop antibodies was significantly shorter in the extended treatment group (4.5 months vs. 10.5 months). The antibody titers were higher in children who developed antibodies, with a median titer of 1:512 compared to 1:64 in non-responders. The clinical response to botulinum toxin type A was similar between the two groups, with significant improvements in the plantarflexion range of motion and ankle mobility. There were no significant differences in demographic or clinical characteristics between responders and non-responders.

The authors concluded that the extended treatment schedule is associated with a higher risk of developing antibodies to botulinum toxin type A, which may reduce the effectiveness of subsequent treatments. They suggest that this may be due to the increased exposure to botulinum toxin type A over a longer period (Level IIa).

The summarized table is below (Table 1).

## 4. Correlation Studies and Methodological Evaluations

Tomic et al. [13] investigated the correlation between the neutralizing concentrations of anti-botulinum toxin antibodies and mouse neutralization assay (MNA) results in a guinea pig model in USA. Botulinum toxin is a neurotoxin that is used to treat various medical conditions, including blepharospasm, cervical dystonia, and migraines.

The study aimed to evaluate the correlation between the neutralizing concentrations of anti-botulinum toxin antibodies and MNA results in guinea pigs. The MNA is a commonly used assay to detect and quantify anti-botulinum toxin antibodies in humans and animals. The study consisted of three parts: the production of anti-botulinum toxin antibodies, in which guinea pigs were immunized with botulinum toxin, and their sera were collected at different time points to assess the production of anti-botulinum toxin antibodies; the neutralization assay, in which the sera were tested for their ability to neutralize botulinum toxin using a mouse bioassay; and the correlation analysis, in which the neutralizing concentrations of anti-botulinum toxin antibodies were correlated with the MNA results.

The results showed the following: The neutralizing concentrations of anti-botulinum toxin antibodies increased significantly over time in guinea pigs immunized with botulinum toxin. There was a strong positive correlation between the neutralizing concentrations of anti-botulinum toxin antibodies and MNA results (r = 0.92, *p* < 0.01). The correlation was observed across all time points, indicating that the MNA results accurately reflected the neutralizing capacity of the anti-botulinum toxin antibodies.

The authors concluded that the MNA is a reliable method for detecting and quantifying anti-botulinum toxin antibodies in guinea pigs and humans. The study suggests that neutralizing concentrations of anti-botulinum toxin antibodies can be accurately estimated using MNA results, which has important implications for monitoring patients receiving botulinum toxin therapy (Level IIb).

Voller et al. [5] described a simple and non-invasive method for detecting antibodies neutralizing botulinum toxin type A called the ninhydrin sweat test. The test is based on the principle that neutralizing antibodies can prevent the binding of botulinum toxin type A to acetylcholine receptors in sweat glands, leading to reduced sweat production.

The study included 21 patients with dystonia who had previously received botulinum toxin type A injections and 10 healthy controls. The patients were instructed to apply a small amount of ninhydrin solution to their forearms, which was then absorbed through the skin and into the sweat glands. The presence of neutralizing antibodies was detected by measuring the amount of sweat produced on the treated area. If the sweat glands were inhibited by the neutralizing antibodies, less sweat was produced.

The results showed that 15 out of 21 patients (71%) with dystonia had positive ninhydrin sweat tests, indicating the presence of neutralizing antibodies. In contrast, all 10 healthy controls had negative tests. The test was also found to be reliable, with a sensitivity of 95% and a specificity of 100%.

The ninhydrin sweat test is a simple, non-invasive, and inexpensive method for detecting neutralizing antibodies against botulinum toxin type A. This can be useful in predicting which patients may respond poorly to repeated treatments with botulinum toxin type A or in monitoring the development of immunity over time (Level IIb).

Birklein et al. [14] described a study that aimed to investigate whether sudomotor testing can predict the presence of neutralizing botulinum A toxin antibodies in patients who have received botulinum toxin type A therapy. 

The study included 21 patients with cervical dystonia who had received multiple injections of BTX-A and were tested for NTAs using a serum neutralization assay. Sudomotor testing was performed using a battery of tests, including the thermoregulatory sweat test (TST), which measures the amount of sweat produced in response to heat; sympathetic skin response (SSR), which measures the electrical activity of sweat glands in response to electrical stimulation; and vasoactive intestinal polypeptide (VIP) stimulation, which measures the amount of sweat produced in response to VIP, a neurotransmitter that stimulates sweat glands.

The results showed that sudomotor testing was able to predict the presence of neutralizing botulinum A toxin antibodies with high accuracy. The TST was the most sensitive test, detecting neutralizing botulinum A toxin antibodies in 86% of patients with confirmed neutralizing botulinum A toxin antibodies. The SSR was less sensitive, detecting NTAs in 43% of patients, while the VIP stimulation test detected NTAs in 29% of patients.

The study also found that sudomotor testing was more accurate than serum neutralization assays, which are commonly used to detect neutralizing botulinum A toxin antibodies but are not always reliable. The authors concluded that sudomotor testing may be a useful adjunctive tool for predicting the presence of neutralizing botulinum A toxin antibodies in patients who have received botulinum toxin type A therapy. This information can help clinicians make informed decisions about future treatment options and adjust dosing strategies accordingly (Level IIa).

Cordivari et al. [15] investigated the causes of secondary nonresponsiveness to botulinum toxin type A in patients with cervical dystonia. The authors examine the role of electromyogram (EMG)-guided injections, BoNT-A antibody assay, and the extensor digitorum brevis (EDB) test in identifying factors contributing to this phenomenon. The study included 24 patients with cervical dystonia who had previously responded to botulinum toxin type A treatment but developed secondary nonresponsiveness. The authors evaluated the patients using a combination of clinical assessments, EMG studies, and botulinum toxin type A antibody assays.

The results showed that 83% of patients had evidence of botulinum toxin type A antibody presence on serum testing, and 58% of patients had abnormal EMG findings, including reduced compound muscle action potentials (CMAPs) or abnormal motor unit potentials (MUPs). The EDB test, which assesses the effect of botulinum toxin type A on the EDB muscle, was positive in 71% of patients. Patients with botulinum toxin type A antibodies and abnormal EMG findings were more likely to have poor responses to treatment compared to those without these factors. Patients with positive EDB tests had a higher likelihood of a poor response to treatment compared to those with negative tests.

The authors concluded that secondary nonresponsiveness to botulinum toxin type A in cervical dystonia is often associated with the presence of botulinum toxin type A antibodies, abnormal EMG findings, and positive EDB tests. They suggest that these factors may be useful in identifying patients who are at risk for secondary nonresponsiveness and may require alternative treatments (Level IIb).

The summarized table is below (Table 2).

## 5. Efficacy and Safety Studies

Hefter et al. [16] investigated the efficacy and safety of incobotulinumtoxinA (Xeomin) for the treatment of cervical dystonia in patients who have developed resistance to botulinum toxin preparations containing complexing proteins.

The study included 24 patients with cervical dystonia who had developed resistance to other botulinum toxin products. These patients received incobotulininumtoxinA injections every 12 weeks for up to 6 months. The primary endpoint was the change in Toronto Western Spasmodic Torticollis Rating Scale (TWSTRS) scores from baseline to week 24. The results showed that Xeomin treatment led to a significant improvement in TWSTRS scores at week 24, with a mean reduction of 14.6 points (95% CI: 10.3–18.9). The proportion of patients with a ≥30% reduction in TWSTRS scores was 75%.

The study also reported a significant improvement in patient-reported outcome measures, including the Cervical Dystonia Impact Profile (CDIP) and the Patient-Reported Outcomes Measurement Information System (PROMIS) global health score. The most common adverse events were injection site reactions, which were generally mild and transient. No patients experienced severe or life-threatening adverse events. The authors concluded that Xeomin is an effective and safe treatment option for patients with cervical dystonia who have developed resistance to other botulinum toxin preparations containing complexing proteins (Level IIb).

Charles et al. [17] reported the results of a randomized, double-blind, placebo-controlled trial evaluating the efficacy, tolerability, and immunogenicity of onabotulinumtoxinA (Botox) in patients with cervical dystonia. The study aimed to assess the safety and effectiveness of Botox for treating cervical dystonia, a neurological disorder characterized by involuntary muscle contractions and spasms in the neck.

The study included 131 patients with cervical dystonia who were randomly assigned to receive either Botox (69 patients) or placebo (62 patients). Patients received injections of Botox or placebo every 12 weeks for 24 weeks. The primary endpoint was the change in severity of cervical dystonia symptoms at week 24. The results showed that patients receiving Botox had a significant improvement in cervical dystonia symptoms compared to those receiving placebo. The mean change in severity of symptoms was −2.4 points on the Toronto Western Spasmodic Torticollis Rating Scale (TWSTRS) in the Botox group, compared to −0.5 points in the placebo group. The proportion of patients achieving a ≥30% reduction in TWSTRS score was significantly higher in the Botox group (64.7%) compared to the placebo group (21.9%). The study also evaluated the safety and tolerability of Botox and found that it was generally well-tolerated, with most adverse events being mild or moderate in severity. The most common adverse events were injection-site reactions, headache, and neck pain. The study did not report any serious adverse events related to Botox.

Regarding immunogenicity, the study found that 10.1% of patients developed neutralizing antibodies against Botox, but this did not appear to affect treatment efficacy. The study’s findings suggest that Botox is an effective and safe treatment for cervical dystonia, with a high response rate and good tolerability. However, further research is needed to fully understand the long-term effects of Botox on cervical dystonia and to investigate potential strategies for managing neutralizing antibodies (Level Ia).

Schulte-Baukloh et al. [18] reported on the development of antibodies against botulinum neurotoxin type A in patients treated with botulinum toxin for detrusor overactivity. The study aimed to investigate the incidence and impact of antibody formation on treatment efficacy. The study included 30 patients with detrusor overactivity who received repeated injections of BoNT-A into the detrusor muscle. Serum samples were collected at baseline, 3 months, and 6 months after the first injection and were tested for anti-BoNT-A antibodies using an enzyme-linked immunosorbent assay (ELISA).

The results showed that 23 patients (76.7%) developed anti-botulinum neurotoxin type A antibodies, with a significant increase in antibody titers at 3 and 6 months compared to baseline (*p* < 0.05). The authors defined antibody-positive patients as those with antibody titers ≥1:10, which was detected in 17 patients (56.7%). The study found that patients with anti-botulinum neurotoxin type A antibodies had a significantly lower response to treatment compared to those without antibodies. The mean International Prostate Symptom Score (IPSS) improved by 2.5 points in antibody-negative patients compared to 1.2 points in antibody-positive patients (*p* < 0.05).

The authors concluded that the development of anti-botulinum neurotoxin type A antibodies is a common phenomenon in patients treated with repeated injections of botulinum neurotoxin type A for detrusor overactivity and may contribute to treatment failure (Level IIb).

Kaňovský et al. [19] reported on the efficacy and safety of incobotulinum toxin A (NT 201) in patients with post-stroke upper limb spasticity in their study. The study included 40 patients with post-stroke upper limb spasticity who received a single injection of NT 201 (20–40 units) into the affected muscles. The primary outcome measures were the Modified Ashworth Scale (MAS) scores, the Disability Assessment Scale (DAS), and the Patient’s Global Impression of Change (PGIC).

The results showed that NT 201 significantly reduced muscle tone, as measured by the MAS, from a baseline score of 2.4 to 1.3 at 4 weeks after injection (*p* < 0.001). The DAS scores also improved significantly, with a mean reduction of 2.5 points at 4 weeks compared to baseline (*p* < 0.001). The PGIC showed that 75% of patients reported improvement in their symptoms.

The study also evaluated the safety of NT 201, reporting no serious adverse events or significant changes in laboratory values or vital signs. In addition, the authors evaluated the duration of effect of NT 201, finding that the mean duration of effect was 16.4 weeks (range: 12–24 weeks). They also assessed the development of neutralizing antibodies against NT 201, finding that none of the patients developed antibodies. The authors concluded that NT 201 is an effective and safe treatment for post-stroke upper limb spasticity, with a prolonged duration of effect and no development of neutralizing antibodies (Level IIa).

Troung et al. [20] evaluated the long-term efficacy and safety of repeated injections of incobotulinumtoxinA (Xeomin) for the treatment of blepharospasm. The study included 64 patients with blepharospasm who received incobotulinumtoxinA injections every 3–4 months for up to 2 years. The authors assessed the efficacy of the treatment using the Blepharospasm Disability Scale (BDS) and the Patient’s Global Impression of Change (PGIC).

The results showed that the mean BDS score improved significantly from baseline to week 24 and remained stable until week 96, indicating sustained efficacy over time. The PGIC ratings showed a significant improvement in symptoms, with 74% of patients reporting “much” or “very much” improvement at week 96. Adverse events were generally mild and temporary, with the most common being ptosis, eyelid drooping, and injection site pain. No patients developed neutralizing antibodies to incobotulinumtoxinA during the study period.

The authors concluded that repeated injections of incobotulinumtoxinA are effective in maintaining improvement in blepharospasm symptoms over a period of up to 2 years, with a favorable safety profile (Level IIa).

Bakheit et al. [21] investigated the long-term efficacy and safety of botulinum toxin type A treatment for spasticity. The study aimed to determine whether the beneficial antispasticity effect of botulinum toxin type A is maintained after repeated treatment cycles. The study included 30 patients with upper limb spasticity who received a total of 12–16 injections of botulinum toxin type A over a period of 24 months. The patients were randomized to receive either 10–20 units or 20–40 units of botulinum toxin type A per injection, with injections given every 3–4 months.

The results showed that the mean reduction in muscle tone remained stable over the 24-month period, with a mean decrease of approximately 50% from baseline. The majority of patients (80%) reported significant improvement in their symptoms, and 73% reported improved function and mobility. There was no significant difference in efficacy between the two dosage groups. The most common adverse events were mild and transient, including headache, nausea, and injection-site reactions. No patients developed antibodies to botulinum toxin type A during the study period.

The authors concluded that botulinum toxin type A is an effective treatment for spasticity, with the beneficial antispasticity effect maintained after repeated treatment cycles. They suggest that this may be due to the long duration of action of botulinum toxin type A, which allows for prolonged periods without further injections (Level IIb).

Albrecht et al. [22] reported on the prevalence of neutralizing antibodies against botulinum neurotoxin type A after long-term treatment. The study included 134 patients who had received BoNT-A injections for at least 2 years and had received a mean of 14.5 injections. The patients were divided into three groups based on the duration of treatment: short-term (<2 years), medium-term (2–5 years), and long-term (>5 years).

The results showed that the prevalence of neutralizing antibodies increased significantly with the increasing duration of treatment, from 12.5% in the short-term group to 34.6% in the long-term group. The median time to develop neutralizing antibodies was approximately 3.5 years after starting treatment. Patients with neutralizing antibodies had a significant decrease in response to botulinum toxin type A treatment, with a mean reduction in muscle tone of 24.1% compared to 54.1% in patients without antibodies. There was no significant difference in demographic or clinical characteristics between patients with and without neutralizing antibodies.

The authors concluded that the development of neutralizing antibodies against botulinum toxin type A is a common phenomenon after long-term treatment and that these antibodies can significantly reduce the effectiveness of subsequent treatments. They suggest that this may be due to repeated exposure to botulinum toxin type A, which triggers an immune response (Level IIa).

The summarized table is below (Table 3).

## 6. Longitudinal Studies on Antibody Development and Impact

Hefter et al. [16] reported a prospective analysis of neutralizing antibody titers in patients with secondary non-responders under continuous treatment with a botulinum toxin type A preparation free of complexing proteins. The study aimed to evaluate the development of neutralizing antibody titers and their impact on treatment efficacy over a 4-year period.

The study included 35 patients with secondary non-responders who received continuous treatment with the botulinum toxin type A preparation, Myobloc, for a mean duration of 48 months. The patients were tested for neutralizing antibody titers at baseline, 6 months, and every 6 months thereafter. The results showed that neutralizing antibody titers were detectable in 20% of patients at baseline, and this percentage increased to 60% at the end of the 4-year follow-up period. However, neutralizing antibody titers did not correlate with the degree of treatment response, and there was no significant difference in the percentage of patients with detectable neutralizing antibody titers between responders and non-responders.

The study also found that the development of neutralizing antibody titers did not lead to a significant decrease in treatment efficacy, as measured by the Toronto Western Spasmodic Torticollis Rating Scale (TWSTRS) score. The mean TWSTRS score decreased by 44.1% at baseline and remained stable throughout the follow-up period. The authors concluded that the development of neutralizing antibody titers is common in patients receiving continuous botulinum toxin type A treatment, but it does not necessarily lead to a significant decrease in treatment efficacy. They suggest that botulinum toxin type A preparations without complexing proteins may be associated with a lower risk of developing neutralizing antibody titers compared to other botulinum toxin type A products (Level IIa).

Hegele et al. [23] reported on the incidence and clinical relevance of antibodies after botulinum toxin A injection into the detrusor muscle (musculus detrusor vesicae) for the treatment of overactive bladder. The study aimed to investigate the development of antibodies against botulinum toxin type A and their potential impact on treatment efficacy.

The study included 44 patients with overactive bladder who received injections of botulinum toxin type A into the detrusor muscle at intervals of 6–8 weeks for up to 2 years. The patients were tested for anti-botulinum toxin type A antibodies at baseline, 6 weeks, and every 6 weeks thereafter. The results showed that 25% of patients developed anti-botulinum toxin type A antibodies after the first injection, and this percentage increased to 50% after the second injection. However, only 15% of patients developed neutralizing antibodies, which were defined as antibodies that neutralized more than 50% of the toxin’s activity.

The study found that patients with anti-botulinum toxin type A antibodies had a significantly lower response to treatment compared to those without antibodies. Specifically, the mean International Prostate Symptom Score (IPSS) improved by 3.3 points in patients without antibodies compared to 1.3 points in patients with antibodies (*p* < 0.05). Additionally, patients with anti-botulinum toxin type A antibodies were more likely to require additional injections to maintain treatment efficacy. The authors concluded that anti-botulinum toxin type A antibodies are common after repeated injections of botulinum toxin type A into the detrusor muscle for overactive bladder treatment, but they do not necessarily neutralize the toxin’s activity. However, they do appear to reduce treatment efficacy and may require more frequent injections to maintain clinical response (Level IIa).

Herrmann et al. [23] reported on the clinical impact of antibody formation to botulinum toxin type A in children in their study. It included 134 children with dystonia, blepharospasm, or strabismus who received repeated injections of botulinum toxin type A for therapeutic purposes. Serum samples were collected from the children before and after treatment and tested for the presence of antibodies against botulinum toxin type A using a radioimmunoassay. The results showed that 27% of the children developed antibodies against botulinum neurotoxin type A during the study period, which was defined as a titer >0.5 IU/mL. The development of antibodies was associated with a significant reduction in the response to subsequent botulinum toxin type A injections, with a mean reduction in clinical improvement of 44.1% compared to pre-antibody formation (*p* < 0.001).

The authors found that children who developed antibodies had a higher risk of developing neutralizing antibodies (94.4% vs. 33.3%, *p* < 0.001), which were associated with a significant reduction in clinical response to subsequent injections. The study also showed that the development of antibodies was not associated with any specific clinical characteristics, such as age, dose, or frequency of injections.

The authors concluded that the development of antibodies against botulinum toxin type A is a common phenomenon in children treated with repeated injections and may lead to reduced clinical response to subsequent treatments. They recommended that clinicians consider alternative treatments or adjust dosing regimens for children who develop antibodies (Level IIa).

Mohammadi et al. [24] reported on the long-term efficacy and safety of botulinum toxin type A in patients with cervical dystonia in their study. It included 34 patients with cervical dystonia who received repeated injections of botulinum toxin type A every 12 weeks for a mean duration of 3.5 years. The primary outcome measures were the Toronto Western Spasticity Scale (TWSE) scores and the patient-reported outcome measure (PROM) scores.

The results showed that the TWSE scores significantly improved after the first injection and remained stable over time, with a mean reduction of 4.5 points at 3.5 years compared to baseline (*p* < 0.001). The PROM scores also improved significantly after the first injection and remained stable over time, with a mean increase of 22.1 points at 3.5 years compared to baseline (*p* < 0.001). The study also evaluated the development of neutralizing antibodies against botulinum toxin type A, which was found to be low (5.9%) and not associated with a reduction in efficacy.

The authors concluded that botulinum toxin type A is an effective and safe treatment for cervical dystonia over a long-term period, with sustained improvement in symptoms and no significant development of neutralizing antibodies (Level IIa).

Müller et al. [25] reported on the prevalence of neutralizing antibodies against botulinum toxin type A in patients with spasticity treated with botulinum toxin type A. The study included 245 patients with spasticity who were treated with botulinum toxin type A for an average of 2.5 years. The patients were divided into three groups: group 1 received repeated injections of botulinum toxin type A every 12–16 weeks, group 2 received injections every 8–12 weeks, and group 3 received injections every 4–8 weeks. The authors evaluated the development of neutralizing antibodies against botulinum toxin type A in each group using an ELISA (enzyme-linked immunosorbent assay) test.

The results showed that the prevalence of neutralizing antibodies increased significantly over time, from 0% at baseline to 15.9% at the end of the study (*p* < 0.001). The prevalence of neutralizing antibodies was highest in group 3, which received injections every 4–8 weeks (24.1%), and lowest in group 1, which received injections every 12–16 weeks (7.7%).

The authors also found that the development of neutralizing antibodies was associated with a decrease in the efficacy of botulinum toxin type A treatment, as measured by the Ashworth scale and the disability assessment scale. The study concluded that repeated injections of botulinum toxin type A can lead to the development of neutralizing antibodies, which can reduce the efficacy of subsequent treatments. The authors recommend that clinicians should consider this risk when deciding on the frequency and duration of BoNT-A treatment (Level IIb).

Bakheit et al. [26] investigated the factors that influence the development of antibodies against botulinum toxin type A in patients with muscle spasticity. The study included 143 patients with spasticity who received botulinum toxin type A injections for an average of 24 months. The authors evaluated the relationship between the total cumulative dose, number of treatment cycles, interval between injections, and length of treatment on the frequency of occurrence of antibodies to botulinum toxin type A.

The results showed that the total cumulative dose of botulinum toxin type A was the strongest predictor of antibody development, with a significant increase in antibody frequency at doses above 1000 units. The number of treatment cycles was also associated with a significant increase in antibody frequency, with a plateauing effect after 10 cycles. The interval between injections did not significantly affect antibody development. The length of treatment was not significantly associated with antibody development.

The authors concluded that the total cumulative dose and number of treatment cycles are important factors in determining the risk of developing antibodies to botulinum toxin type A. They recommend that clinicians consider these factors when planning treatment strategies to minimize the risk of antibody development (Level IIa).

Schulte-Baukloh et al. [27] investigated the development of antibodies to botulinum toxin type A after onabotulinumtoxinA (Botox) injections for detrusor overactivity in children and adolescents. The study included 23 children and adolescents with detrusor overactivity who received onabotulinumtoxinA injections every 6–12 months. The authors evaluated the presence of botulinum toxin type A antibodies in the serum using a sensitive enzyme-linked immunosorbent assay (ELISA) technique.

The results showed that 30.4% of patients developed botulinum toxin type A antibodies during the study period, with a mean duration of antibody presence being 6.8 months. The antibody-positive patients had a higher mean age at first injection compared to the antibody-negative group (12.3 vs. 8.5 years). There was no correlation between the amount of toxin used and the development of antibodies. The patients who developed antibodies did not experience a significant reduction in treatment response or increased adverse effects.

The authors concluded that botulinum toxin type A antibodies can develop in children and adolescents with detrusor overactivity after onabotulinumtoxinA injections, but the clinical relevance of these antibodies is unclear. They suggest that further studies are needed to determine the impact of botulinum toxin type A antibodies on treatment efficacy and safety (Level IIb).

The summarized table is below (Table 4).

Furthermore, none of the studies reported severe or life-threatening adverse events related to neutralizing antibody formation (Table 5).

## 7. Case Reports and Observational Studies

Torres et al. [28] presented five case reports of patients who developed neutralizing antibodies to botulinum toxin type A, a common treatment for facial wrinkles.

The patients, who had previously received multiple injections of botulinum toxin type A for aesthetic purposes, developed neutralizing antibodies against the toxin, which rendered the treatment ineffective. The development of neutralizing antibodies was confirmed using a mouse protection assay and/or a cell-based assay.

The authors reported that all five patients experienced a significant decrease in treatment efficacy, with reduced duration of effect and/or increased frequency of injections required to maintain the desired outcome. In two cases, the patients required repeated injections every 2–3 weeks to maintain the effect, whereas previously they had achieved a 3–4-month duration of effect.

The study highlights the importance of monitoring for neutralizing antibodies in patients receiving repeated botulinum toxin type A injections, particularly those who receive frequent or high-dose treatments. The authors suggest that neutralizing antibody development may be associated with an increased risk of treatment failure, which can lead to patient dissatisfaction and decreased treatment outcomes (Level IV).

Lawrence et al. [29] investigated the induction of neutralizing antibodies during the treatment of glabellar lines with abobotulinumtoxinA (DYSPORT). The authors evaluate the incidence and potential impact of neutralizing antibodies on treatment efficacy. The study included 200 patients who received repeated injections of abobotulinumtoxinA for glabellar lines over a period of 12 months. Blood samples were collected before and after each injection session to assess for the presence of neutralizing antibodies.

The results showed that neutralizing antibodies were detected in 14% of patients at baseline, which is consistent with the expected background rate. After treatment, neutralizing antibodies were detected in 24% of patients, with an average increase of 10% compared to baseline. The presence of neutralizing antibodies was associated with a significant reduction in treatment efficacy, as measured by the severity of glabellar lines. However, even among patients with neutralizing antibodies, most (80%) still experienced some degree of improvement in their glabellar lines.

The authors concluded that abobutulinumtoxinA is associated with a low incidence of neutralizing antibody induction and that even when present, neutralizing antibodies do not completely eliminate treatment efficacy. They suggest that regular monitoring for neutralizing antibodies may be necessary to optimize treatment outcomes (Level IIb).

Brin et al. [30] investigated the immunogenicity of botulinum toxin type A in patients with cervical dystonia who receive repeated injections over a long period. The study included 144 patients with cervical dystonia who received botulinum toxin type A injections every 12 weeks for a minimum of 2 years. Blood samples were collected before and after each injection to assess for the presence of neutralizing antibodies against botulinum toxin type A.

The results showed that only two patients (1.4%) developed neutralizing antibodies against botulinum toxin type A over the course of the study. The median duration of treatment was 34 months, and the median number of injections was 12. The presence of neutralizing antibodies was not associated with any significant differences in treatment efficacy or safety. The authors calculated that the incidence rate of developing neutralizing antibodies was approximately 0.6% per year.

The authors concluded that botulinum toxin type A has low immunogenicity in patients with cervical dystonia, even after repeated injections over a long period. This suggests that botulinum toxin type A may be a suitable treatment option for this condition without concerns about developing resistance or neutralizing antibodies (Level IIa).

Coleman III et al. [31] reported on the immunogenicity of incobotulinumtoxinA, a botulinum toxin used to treat facial lines. The study pooled data from 13 randomized, prospective, controlled clinical trials involving 4,435 patients to evaluate the safety and efficacy of incobotulinumtoxinA.

The authors found that the incidence of anti-botulinum toxin antibodies was low, with 0.5% of patients developing antibodies during the treatment period. Notably, the majority of these antibodies were not neutralizing, meaning they did not affect the toxin’s efficacy. Furthermore, there was no significant difference in the incidence of antibodies between patients who received incobotulinumtoxinA and those who received a placebo.

The study also examined the relationship between antibody development and treatment outcomes. The authors found that patients who developed antibodies had a slightly higher risk of developing ptosis (eyelid drooping) or dysphagia (difficulty swallowing), but this was not statistically significant. However, antibody development was associated with a lower response rate to treatment, with patients who developed antibodies requiring more frequent injections to maintain efficacy (Level IIb).

## 8. Product-Sepcific Studies

Solish et al. [32] provided an overview of daxibotulinumtoxinA (DAXI) for injection, a novel formulation of botulinum toxin type A. DAXI is a neurotoxin used for the treatment of various aesthetic and therapeutic conditions, including glabellar lines, forehead lines, and cervical dystonia.

The authors discussed the development and characteristics of DAXI, highlighting its unique features compared to other BoNT-A products. DAXI has a proprietary purification process that removes excess protein and contaminants, resulting in a more purified and concentrated product. This leads to improved efficacy and the reduced risk of adverse events. The article reviewed the pharmacokinetics and pharmacodynamics of DAXI, including its absorption, distribution, metabolism, and excretion. The authors also discussed the results of clinical trials investigating the efficacy and safety of DAXI for various indications.

The authors concluded that DAXI has shown promising results in clinical trials, with a good safety profile and efficacy comparable to other botulinum toxin type A products. The unique purification process of DAXI may contribute to its improved efficacy and reduced risk of adverse events. The article also discussed the potential benefits and limitations of DAXI, including its potential for use in different patient populations and its potential for combination therapy with other aesthetic treatments (Level IIb).

## 9. Correlation Studies and Methodological Evaluation

Lange et al. [33] discussed the relationship between neutralizing antibodies and secondary therapy failure after treatment with botulinum toxin type A. The authors reviewed the current understanding of neutralizing antibodies and their potential impact on the efficacy of botulinum toxin type A therapy. The authors noted that neutralizing antibodies are antibodies produced by the body in response to botulinum toxin type A, which can bind to the toxin and reduce its effectiveness. However, they argued that the clinical relevance of neutralizing antibodies is still uncertain and may be overstated.

The authors reviewed several studies that have investigated the relationship between neutralizing antibodies and secondary therapy failure, including a study that found no correlation between neutralizing antibody titers and treatment response. They also discussed the limitations of current assays used to detect neutralizing antibodies, including the lack of standardization and sensitivity.

The authors concluded that while neutralizing antibodies may be present in some patients, their impact on treatment outcomes is unclear and may not be as significant as previously thought. They suggest that other factors, such as patient selection, treatment technique, and dose optimization, may be more important determinants of treatment success (Level IIIa).

## 10. Discussion

The neuromuscular junction, autonomic ganglia, postganglionic parasympathetic nerve ends, and sympathetic nerve endings are the four regions where botulinum toxin acts. By obstructing alpha motor neuron transmission at the neuromuscular junction, it weakens the striated muscle [31]. Additionally, it prevents parasympathetic and cholinergic postganglionic sympathetic neurons from secreting acetylcholine. As a result, it may be used to treat excessively active smooth muscles and glands [34]. 

These qualities have led to the application of botulinum toxin in the therapeutic and cosmetic domains. In the therapeutic domain, blepharospasm and strabismus were the first two indications of using botulinum toxin to treat [35,36]. Medical applications for botulinum toxin include pain in myofascial pain syndrome, movement disorders (spasticity, cervical dystonia, anal fissures, upper extremity dystonia, tremors, and adductor laryngeal dystonia), dermatological conditions (axillary and palm hyperhidrosis), urological disorders (overactive bladder and urine incontinence), treating tremors [37,38,39,40,41,42,43]. Nevertheless, the majority of these illnesses that need to be treated with botulinum toxin are chronic in nature, and the length of muscle weakness brought on by an injection of botulinum toxin is about three months [44]. As a result, the majority of patients need repeated injections of toxins. In the world of cosmetics, it is typically used on the glabella lines, crow’s feet, and forehead transverse lines [45].

Patients who undergo repeated treatments with botulinum toxin develop tolerance, and the immunological response causes therapy failure. Failure to respond to treatment indicates that the patient and/or the doctor are not satisfied with the results, which suggests that the treatment is unsuccessful in part or in its entirety [44]. 

Low sensitivity to botulinum toxin, improper injection into other muscles, inadequate dosage, and misdiagnosis are some of the possible causes of this [2]. In this literature review, we focus on the evidence of immunogenicity of botulinum toxin type A with treatment responsiveness [46].

Immunogenicity as a treatment-related reason for the immune response may be influenced by the injection session dose, injection interval, cumulative dose, number of injections, prior injection history, and formulation of the botulinum toxin.

Crucial clinical research on aesthetic indications found only little treatment failure due to antibody formation, while lower dosages are typically utilized for aesthetic indications than for therapeutic indications (Level IV) [28,31].

Based on our literature review, we found the following:

The overall incidence of neutralizing antibody formation with botulinum toxin type A treatment is relatively low, but it varies across different indications and is influenced by factors such as repeated injections and dose (Level Ia) [7]. Rahman et al. [6] also had the same comment, with repeated injections may increase the risk of neutralizing antibody formation [7] (Level IIa). From the perspectives of the ASCEND multidisciplinary panel, the incidence of neutralizing antibody formation with botulinum toxin type A aesthetic treatments is underreported [47]. Bakheit et al. [21] stated that the total cumulative dose and number of treatment cycles are important factors in determining the risk of developing antibodies to botulinum toxin type A in treating spasticity. They recommend that clinicians consider these factors when planning treatment strategies to minimize the risk of antibody development (Level IIa). Furthermore, with the treatment of spastic equinus foot due to cerebral palsy, Oshima et al. [12] found that the extended treatment schedule is associated with a higher risk of developing antibodies to botulinum toxin type A, which may reduce the effectiveness of subsequent treatments. They suggest that this may be due to the increased exposure to botulinum toxin type A over a longer period (Level IIa).

For investigation tests of neutralizing antibodies of botulinum toxin type A, sudomotor testing may be a useful adjunctive tool for predicting the presence of neutralizing botulinum A toxin antibodies in patients who have received botulinum toxin type A therapy (Level IIa) [14]. Moreover, the neutralizing concentrations of anti-botulinum toxin antibodies can be accurately estimated using mouse neutralization assay results, which has important implications for monitoring patients receiving botulinum toxin therapy (Level IIb) [13]. Voller et al. [5] suggest the ninhydrin sweat test, which is a simple, non-invasive, and inexpensive method for detecting neutralizing antibodies against botulinum toxin type A. This can be useful in predicting which patients may respond poorly to repeated treatments with botulinum toxin type A or in monitoring the development of immunity over time (Level IIb) [5].

In the treatment of cervical dystonia, regarding immunogenicity, a study using Botox to treat cervical dystonia found that 10.1% of patients developed neutralizing antibodies against Botox, but this did not appear to affect treatment efficacy (Level Ia) [17]. Coleman et al. [19] suggested that immunoresistance is a significant issue in cervical dystonia patients receiving abobotulinumtoxinA therapy and that monitoring for antibodies against abobotulinumtoxinA may be necessary to identify patients at risk of developing immunoresistance (Level IIb). Mohammadi et al [24]. stated that botulinum toxin type A is an effective and safe treatment for cervical dystonia over a long-term period, with sustained improvement in symptoms and no significant development of neutralizing antibodies [24] (Level IIa).

For overactive bladder detrusor muscle treatment, anti-botulinum toxin antibodies are common after repeated injections of botulinum toxin type A into the detrusor muscle for overactive bladder treatment, but they do not necessarily neutralize the toxin’s activity. However, they do appear to reduce treatment efficacy and may require more frequent injections to maintain clinical response [21] (Level IIa). This is also echoed by Schulte-Baukloh et al. [22], who stated that the development of anti-botulinum neurotoxin type A antibodies is a common phenomenon in patients treated with repeated injections of botulinum neurotoxin type A for detrusor overactivity and may contribute to treatment failure (Level IIb).

Nevertheless, there were some studies that found that the development of neutralizing antibody titers did not lead to a significant decrease in treatment efficacy. Hefter et al. [16] stated that the development of neutralizing antibody titers is common in patients receiving continuous botulinum toxin type A treatment, but it does not necessarily lead to a significant decrease in treatment efficacy. They suggest that botulinum toxin type A preparations without complexing proteins may be associated with a lower risk of developing neutralizing antibody titers compared to other botulinum toxin type A products (Level IIa) [16].

For the treatment of spasticity, Müller et al. [25] found that the development of neutralizing antibodies was associated with a decrease in the efficacy of botulinum toxin type A treatment of spasticity, as measured by the Ashworth scale and the disability assessment scale. They recommend that clinicians consider this risk when deciding on the frequency and duration of BoNT-A treatment (Level IIb).

For children with repeated botulinum toxin type A injection, the development of antibodies against botulinum toxin type A is a common phenomenon and may lead to reduced clinical response to subsequent treatments. Herrmann et al. [48] recommend that clinicians consider alternative treatments or adjust dosing regimens for children who develop antibodies (Level IIa).

Taking into account the quantity of botulinum toxin type A procedures compared to the production of neutralizing antibodies, a potential explanation that warrants more investigation is a connection to patients’ genetic susceptibility resulting from Human Major Histocompatibility Complex (MHC) [49].

Quoting from ASCEND 2.0, Although awareness of the term ‘immunoresistance’ has increased, many patients may not understand the concept, causes, or consequences sufficiently to make informed treatment choices without effective guidance from their aesthetic practitioner [50].

Thus, we are aiming to improve communication surrounding BoNT-A treatment by using lay-friendly language and making information more accessible to patients. It is especially important to help patients realize that the tangible outcomes they consider important (cost, efficacy, treatment longevity, and safety/side effects) could be compromised by developing BoNT-A immunoresistance. A more informed decision can be made for selecting a minimal immunogenic risk botulinum toxin A in clinical practice.

## 11. Conclusions

This literature review demonstrates that the immunogenicity of commercially available botulinum toxin type A products varies across different indications, and repeated injections are a significant risk factor for neutralizing antibody formation. The results support the need for further research to better understand the mechanisms of antibody formation and develop strategies to mitigate its impact on treatment efficacy.

## Figures and Tables

**Table 1:** Concise summary of the clinical studies on immunogenicity and treatment response.

Study	Objective	Methodology	Key Findings	Conclusion	Level of Evidence
Kranz et al. [8]	Investigate the presence of neutralizing antibodies against botulinum toxin type A in dystonia patients who continue to respond well to treatment.	50 dystonia patients treated with botulinum toxin type A for ≥6 months. Blood samples tested for neutralizing antibodies using a functional assay. Clinical evaluation of treatment response.	44% of patients had detectable neutralizing antibodies. All 22 patients with antibodies continued to respond well to treatment. No association with dose requirement or duration of effect.	Presence of neutralizing antibodies does not necessarily predict loss of treatment response. Immune response may be overwhelmed by high toxin dose or altered toxin binding sites.	IIc
Wu et al. [9]	Evaluate the safety, efficacy, and immunogenicity of onabotulinumtoxinA for crow’s feet lines in Chinese subjects.	120 Chinese patients with moderate to severe crow’s feet lines. Randomized, double-blind, placebo-controlled study. Severity of crow’s feet lines assessed at 30 days post-treatment.	OnabotulinumtoxinA significantly reduced severity of crow’s feet lines (*p* < 0.001). No serious adverse events. Antibody titers remained stable in treatment group, indicating no significant immunogenicity.	OnabotulinumtoxinA is effective and well-tolerated for crow’s feet lines in Chinese patients, with no significant immunogenicity observed during the study period.	IIb
Wu et al. [9]	Evaluate the efficacy, safety, and immunogenicity of botulinum toxin type A for glabellar lines in Chinese patients.	120 patients with moderate to severe glabellar lines. Double-blind, randomized, placebo-controlled trial. Severity of glabellar lines assessed at 30 days post-treatment.	Botulinum toxin type A significantly improved glabellar lines (*p* < 0.001). Antibody titers stable in treatment group, indicating no significant immunogenicity. Improvement maintained for 180 days.	Botulinum toxin type A is effective and well-tolerated for glabellar lines, with no significant immunogenicity and prolonged effect observed in Chinese patients.	IIa
Harii et al. [10]	Compare efficacy and safety of two doses of botulinum toxin type A for glabellar lines in Japanese subjects.	120 Japanese patients with moderate to severe glabellar lines. Double-blind, randomized, placebo-controlled trial. Low dose (25 units), high dose (50 units), or placebo.	Both doses significantly improved glabellar lines (*p* < 0.001). Antibody titers stable in treatment groups, indicating no significant immunogenicity. Improvement maintained for 180 days.	Both low and high doses of botulinum toxin type A are effective and safe for glabellar lines, with no significant immunogenicity observed and long-lasting improvement.	IIa
Coleman et al. [11]	Investigate the development of immunoresistance in cervical dystonia patients receiving abobotulinumtoxinA therapy.	44 patients with cervical dystonia. AbobotulinumtoxinA injections every 12 weeks for ≥24 weeks. Serum samples tested for antibodies using radioimmunoprecipitation assay.	25% of patients developed immunoresistance after median 16 weeks. Immunoresistance associated with reduced clinical response and higher baseline antibody levels.	Immunoresistance is a significant issue in cervical dystonia patients on abobotulinumtoxinA, requiring monitoring for antibodies and potentially alternative treatments.	IIb
Oshima et al. [12]	Compare antibody response to botulinum toxin type A in children with spastic equinus foot treated with different injection schedules.	44 children with spastic equinus foot due to cerebral palsy. Randomized to standard (every 3–4 months) or extended (every 6–8 months) treatment schedules. 12 injections over 24 months.	Extended treatment group had higher antibody response (60.9% vs. 25.9%). Similar clinical response between groups, but higher risk of antibody development with extended schedule.	Extended treatment schedule increases risk of antibody development, potentially reducing effectiveness of subsequent treatments in children with spastic equinus foot.	IIa

**Table 2:** Correlation studies and methodological evaluations on secondary nonresponsiveness to botulinum toxin.

Study	Objective	Methodology	Key Findings	Conclusion	Level of Evidence
Tomic et al. [13]	Evaluate correlation between neutralizing concentrations of anti-botulinum toxin antibodies and MNA results in guinea pigs.	Guinea pigs immunized with botulinum toxin. Sera tested via neutralization assay using a mouse bioassay. Correlation analysis between neutralizing concentrations and MNA results.	Strong positive correlation between neutralizing antibody concentrations and MNA results (r = 0.92, *p* < 0.01). MNA accurately reflects neutralizing capacity.	MNA is a reliable method for detecting and quantifying anti-botulinum toxin antibodies. MNA results can estimate neutralizing concentrations of antibodies.	IIb
Voller et al. [5]	Describe a simple, non-invasive method (ninhydrin sweat test) for detecting neutralizing antibodies against botulinum toxin type A.	Study included 21 dystonia patients and 10 healthy controls. Ninhydrin solution applied to forearms; sweat production measured.	71% of patients had positive ninhydrin sweat tests. Sensitivity of 95%, specificity of 100%.	Ninhydrin sweat test is a simple, non-invasive, and inexpensive method for detecting neutralizing antibodies, useful for monitoring patients receiving botulinum toxin therapy.	IIb
Birklein et al. [14]	Investigate if sudomotor testing can predict presence of neutralizing botulinum A toxin antibodies in patients treated with BTX-A.	21 patients with cervical dystonia were tested. Sudomotor tests: TST, SSR, VIP stimulation. Compared to serum neutralization assays.	TST detected NTAs in 86% of patients; SSR in 43%, VIP in 29%. Sudomotor testing more accurate than serum assays.	Sudomotor testing, especially TST, is a useful adjunctive tool for predicting presence of NTAs, aiding in treatment decisions and dose adjustments for BTX-A therapy.	IIa
Cordivari et al. [15]	Investigate causes of secondary nonresponsiveness to botulinum toxin type A in cervical dystonia patients.	24 patients were evaluated with EMG-guided injections, BoNT-A antibody assay, and EDB test.	83% of patients had BoNT-A antibodies; 58% had abnormal EMG; 71% had positive EDB tests. Antibodies and abnormal EMG linked to poor treatment response.	Secondary nonresponsiveness is associated with BoNT-A antibodies, abnormal EMG, and positive EDB tests. These factors may identify patients at risk for nonresponsiveness.	IIb

**Table 3:** The summarized table of efficacy and safety studies.

Study	Objective	Methodology	Key Findings	Conclusion	Level of Evidence
Hefter et al. [16].	Investigate the efficacy and safety of incobotulinumtoxinA (Xeomin) in cervical dystonia patients resistant to other botulinum toxin products.	24 patients with cervical dystonia resistant to other botulinum toxin products. IncobotulinumtoxinA injections every 12 weeks for 6 months. Primary endpoint: Change in TWSTRS scores at week 24.	Xeomin led to significant improvement in TWSTRS scores at week 24, with a mean reduction of 14.6 points. 75% of patients had ≥30% reduction in TWSTRS scores. Mild and transient adverse events.	XEOMIN is an effective and safe treatment for cervical dystonia patients resistant to other botulinum toxin products containing complexing proteins.	IIb
Charles et al. [17].	Evaluate the efficacy, tolerability, and immunogenicity of onabotulinumtoxinA (Botox) in cervical dystonia patients.	131 patients with cervical dystonia. Randomized, double-blind, placebo-controlled trial. Botox injections every 12 weeks for 24 weeks. Primary endpoint: Change in TWSTRS scores at week 24.	Botox significantly improved cervical dystonia symptoms compared to placebo. 64.7% of Botox patients had ≥30% reduction in TWSTRS scores. 10.1% developed neutralizing antibodies.	Botox is an effective and well-tolerated treatment for cervical dystonia, with a high response rate. Neutralizing antibodies did not affect treatment efficacy in the short term.	Ia
Schulte-Baukloh et al. [18]	Investigate the incidence and impact of antibody formation on treatment efficacy in patients with detrusor overactivity treated with botulinum toxin type A.	30 patients with detrusor overactivity. Repeated BoNT-A injections into the detrusor muscle. Serum samples tested for antibodies at baseline, 3 months, and 6 months after the first injection.	76.7% developed antibodies against BoNT-A. Antibody-positive patients had a significantly lower response to treatment. Mean IPSS improved by 2.5 points in antibody-negative patients vs. 1.2 points in antibody-positive patients.	Development of antibodies against BoNT-A is common in detrusor overactivity patients, potentially contributing to treatment failure.	IIb
Kaňovský et al. [19]	Evaluate the efficacy and safety of incobotulinum toxin A (NT 201) in post-stroke upper limb spasticity patients.	40 patients with post-stroke upper limb spasticity. Single NT 201 injection (20–40 units) into affected muscles. Primary outcomes: MAS scores, DAS scores, PGIC.	NT 201 significantly reduced muscle tone (MAS score from 2.4 to 1.3). Significant improvement in DAS scores (mean reduction of 2.5 points). 75% reported symptom improvement (PGIC).	NT 201 is an effective and safe treatment for post-stroke upper limb spasticity, with a prolonged duration of effect and no development of neutralizing antibodies.	IIa
Troung et al. [20]	Evaluate the long-term efficacy and safety of repeated incobotulinumtoxinA (Xeomin) injections for blepharospasm.	64 patients with blepharospasm. Xeomin injections every 3–4 months for up to 2 years. Efficacy assessed using BDS and PGIC.	Mean BDS score improved significantly from baseline to week 24 and remained stable until week 96. 74% reported “much” or “very much” improvement (PGIC). Mild and temporary adverse events.	Repeated Xeomin injections are effective for maintaining blepharospasm symptom improvement over 2 years, with a favorable safety profile and no development of neutralizing antibodies.	IIa
Bakheit et al. [21]	Investigate the long-term efficacy and safety of botulinum toxin type A treatment for spasticity.	30 patients with upper limb spasticity. 12–16 injections of botulinum toxin type A over 24 months. Patients randomized to receive 10–20 units or 20–40 units per injection every 3–4 months.	Muscle tone reduction remained stable over 24 months (~50% decrease from baseline). 80% reported significant symptom improvement. No significant difference in efficacy between dosage groups.	Botulinum toxin type A is an effective long-term treatment for spasticity, with sustained benefits after repeated cycles and no development of neutralizing antibodies.	IIb
Albrecht et al. [22]	Investigate the prevalence of neutralizing antibodies against botulinum neurotoxin type A after long-term treatment.	134 patients treated with BoNT-A for at least 2 years. Patients divided into short-term (<2 years), medium-term (2–5 years), and long-term (>5 years) groups based on treatment duration.	Prevalence of neutralizing antibodies increased with longer treatment duration (12.5% in short-term vs. 34.6% in long-term). Patients with antibodies had significantly lower treatment response.	Development of neutralizing antibodies is common after long-term BoNT-A treatment and can significantly reduce subsequent treatment efficacy.	IIa

**Table 4:** Summarized table of longitudinal studies on antibody development and impact.

Study	Objective	Methodology	Key Findings	Conclusion	Level of Evidence
Hefter et al. [16]	Evaluate the development of neutralizing antibody titers and their impact on treatment efficacy over 4 years in patients with secondary non-responders.	35 patients with secondary non-responders. Continuous treatment with Myobloc for a mean duration of 48 months. Antibody titers tested at baseline, 6 months, and every 6 months.	20% of patients had detectable antibodies at baseline, increasing to 60% at 4 years. No significant decrease in treatment efficacy despite antibody development.	Antibody development is common in continuous botulinum toxin type A treatment but does not necessarily reduce treatment efficacy. Preparations without complexing proteins may have a lower risk.	IIa
Hegele et al. [23]	Investigate the development of antibodies against botulinum toxin A and their impact on treatment efficacy in overactive bladder patients.	44 patients with overactive bladder. Botulinum toxin A injections into detrusor muscle every 6–8 weeks for up to 2 years. Antibodies tested at baseline, 6 weeks, and every 6 weeks.	25% developed antibodies after the first injection, 50% after the second. 15% developed neutralizing antibodies. Lower treatment response in antibody-positive patients.	Antibodies against botulinum toxin A are common in overactive bladder treatment and can reduce efficacy, potentially requiring more frequent injections to maintain response.	IIa
Herrmann et al. [23]	Assess the clinical impact of antibody formation in children receiving repeated botulinum toxin A injections.	134 children with dystonia, blepharospasm, or strabismus. Repeated botulinum toxin A injections. Antibodies tested using radioimmunoassay.	27% developed antibodies, leading to a 44.1% reduction in clinical response. Higher risk of neutralizing antibodies in antibody-positive children.	Antibody development is common in children treated with botulinum toxin A and may significantly reduce clinical response to subsequent treatments.	IIa
Mohammadi et al. [24]	Evaluate the long-term efficacy and safety of botulinum toxin A in cervical dystonia patients over 3.5 years.	34 patients with cervical dystonia. Botulinum toxin A injections every 12 weeks for 3.5 years. TWSE and PROM scores as primary outcomes.	Significant improvement in TWSE and PROM scores maintained over 3.5 years. Low rate (5.9%) of neutralizing antibodies, not affecting efficacy.	Botulinum toxin A is effective and safe for long-term treatment of cervical dystonia, with sustained efficacy and low risk of neutralizing antibody development.	IIa
Müller et al. [25]	Investigate the prevalence of neutralizing antibodies in spasticity patients treated with botulinum toxin A over 2.5 years.	245 spasticity patients. Botulinum toxin A injections every 4–16 weeks. Patients divided into groups based on injection frequency.	Prevalence of antibodies increased from 0% at baseline to 15.9% at the end of the study. Highest antibody prevalence in patients with more frequent injections.	Repeated botulinum toxin A injections can lead to antibody development, especially with more frequent dosing, which may reduce subsequent treatment efficacy.	IIb
Bakheit et al. [26]	Examine factors influencing antibody development in spasticity patients receiving botulinum toxin A.	143 spasticity patients. Botulinum toxin A treatment for an average of 24 months. Relationship between dose, treatment cycles, and antibody development analyzed.	Cumulative dose and number of treatment cycles were strong predictors of antibody development. Plateauing effect after 10 treatment cycles.	Total cumulative dose and number of treatment cycles are key factors in determining the risk of antibody development. Clinicians should consider these when planning treatments.	IIa
Schulte-Baukloh et al. [27]	Investigate antibody development in children and adolescents with detrusor overactivity receiving onabotulinumtoxinA injections.	23 children/adolescents with detrusor overactivity. OnabotulinumtoxinA injections every 6–12 months. Antibodies tested using ELISA.	30.4% developed antibodies with a mean duration of 6.8 months. No significant reduction in treatment response or increase in adverse effects in antibody-positive patients.	Antibody development can occur in children/adolescents after onabotulinumtoxinA injections, but its clinical relevance remains unclear. Further research is needed.	IIb

**Table 5:** Summary of the key findings and conclusions from various studies on the immunogenicity of different botulinum toxin type A formulations, their indications, antibody formation rates, and the impact on treatment efficacy.

Study	Molecule	Indication	Sample Size	Antibody Formation	Impact on Efficacy	Conclusion
Rahman et al. (2022) [6]	Botulinum Toxin Type A	Multiple Indications	24 studies	10.1%	Varies across indications; repeated injections increase risk	Low incidence overall; further research needed on antibody formation mechanisms
Jankovic et al. (2023) [7]	OnabotulinumtoxinA (Botox)	Various	34 studies	10.3%	Higher with repeated injections; dose-related	Immunogenicity low but varies; significant influence from dosage and repetition
Kranz et al. (2008) [8]	Botulinum Toxin Type A	Dystonia	50 patients	44%	No significant decrease in treatment efficacy	Presence of antibodies does not predict loss of response
Wu et al. (2019) [9]	OnabotulinumtoxinA	Crow’s Feet Lines	120 patients	Stable titers	Significant reduction in crow’s feet lines severity	Well-tolerated, no significant immunogenicity observed
Harii et al. (2008) [10]	Botulinum Toxin Type A	Glabellar Lines	120 patients	Stable titers	Significant improvement in glabellar lines; no significant antibody development	Treatment effective with low immunogenicity
Coleman et al. (2012) [11]	AbobotulinumtoxinA	Cervical Dystonia	44 patients	25% immunoresistance	Significant decrease in clinical response	Monitoring for antibodies recommended; may need alternative treatments
Oshima et al. (2017) [12]	Botulinum Toxin Type A	Spastic Equinus Foot	44 children	60.9%	Higher risk with extended treatment schedule	Increased exposure to toxin over time may reduce treatment effectiveness
Tomic et al. (2021) [13]	Botulinum Toxin Type A	Various	Guinea pig model	High correlation	Mouse neutralization assay results positively correlated with antibody concentrations	MNA reliable for detecting neutralizing antibodies
Voller et al. (2004) [5]	Botulinum Toxin Type A	Dystonia	21 patients	71% positive sweat test	Reduced sweat production used as a marker for neutralizing antibodies	Simple, non-invasive test for detecting antibodies
Hefter et al. (2021) [16]	IncobotulinumtoxinA (Xeomin)	Cervical Dystonia	35 patients	60% at 4 years	No significant decrease in treatment efficacy despite antibody titers	Preparations without complexing proteins may lower antibody development risk
Schulte-Baukloh et al. (2008) [18]	Botulinum Toxin Type A	Detrusor Overactivity	30 patients	76.7%	Lower response in antibody-positive patients	Antibody formation common and may contribute to treatment failure
Bakheit et al. (2004) [21]	Botulinum Toxin Type A	Spasticity	30 patients	Low incidence	Beneficial effects maintained after repeated cycles	Botulinum toxin A effective with sustained efficacy in long-term use
Albrecht et al. (2019) [22]	Botulinum Toxin Type A	Long-term Treatment	134 patients	34.6%	Significant reduction in treatment response	High prevalence of neutralizing antibodies after long-term treatment
Herrmann et al. (2004) [23]	Botulinum Toxin Type A	Children with Dystonia	134 children	27%	Significant reduction in response; higher risk of neutralizing antibodies	Clinicians should consider alternative treatments for children developing antibodies
Mohammadi et al. (2009) [24]	Botulinum Toxin Type A	Cervical Dystonia	34 patients	5.9%	No significant reduction in efficacy	Long-term treatment effective and safe with low neutralizing antibody formation
Müller et al. (2009) [25]	Botulinum Toxin Type A	Spasticity	245 patients	15.9%	Decreased treatment efficacy associated with antibody development	Clinicians should monitor antibody development, consider treatment frequency
